# CircGSAP alleviates pulmonary microvascular endothelial cells dysfunction in pulmonary hypertension via regulating miR-27a-3p/BMPR2 axis

**DOI:** 10.1186/s12931-022-02248-7

**Published:** 2022-11-19

**Authors:** Yuanyuan Sun, Rong Jiang, Xiaoyi Hu, Sugang Gong, Lan Wang, Wenhui Wu, Jinling Li, Xinyang Kang, Shijin Xia, Jinming Liu, Qinhua Zhao, Ping Yuan

**Affiliations:** 1 Department of Cardio-Pulmonary Circulation, Shanghai Pulmonary Hospital, School of Medicine, Tongji University, 507 Zhengmin Road, Yangpu District, Shanghai, 200433 China; 2 Department of Respiratory and Critical Care Medicine, Shandong Provincial Hospital, Shandong First Medical University, Jinan, 250001 China; 3 Department of General Surgery, Jinan Heavy Truck Hospital, Shandong Jinan, 250000 China; 4 Department of Shanghai Institute of Geriatrics, Huadong Hospital, Fudan University, Shanghai, 200040 China

**Keywords:** Idiopathic pulmonary arterial hypertension, Pulmonary microvascular endothelial cells, circGSAP, miR-27a-3p, BMPR2

## Abstract

**Background:**

Our previous study showed that circular RNA-gamma-secretase-activating protein (circGSAP) was down-regulated in pulmonary microvascular endothelial cells (PMECs) in response to hypoxia, and regulated the cell cycle of PMECs via miR-942-5p sponge in pulmonary hypertension (PH). However, the mechanism whether circGSAP affects the dysfunction of PEMCs through other microRNAs (miRNAs) remains largely unknown. Therefore, we aimed to demonstrate the underlying mechanisms of circGSAP regulating PMECs dysfunction by absorbing other miRNAs to regulate target genes in idiopathic pulmonary arterial hypertension (IPAH).

**Methods:**

Quantitative real-time polymerase chain reaction, immunofluorescence staining, Cell Counting Kit-8, Calcein-AM/PI staining, Transwell assay, dual-luciferase reporter assay, and ELISA were used to elucidate the roles of circGSAP.

**Results:**

Here we showed that plasma circGSAP levels were significantly decreased in patients with IPAH and associated with poor outcomes. In vivo, circGSAP overexpression improved survival, and alleviated pulmonary vascular remodeling of monocrotaline-induced PH (MCT-PH) rats. In vitro, circGSAP overexpression inhibited hypoxia-induced PMECs proliferation, migration and increased mortality by absorbing miR-27a-3p. BMPR2 was identified as a miR-27a-3p target gene. BMPR2 silencing ameliorated the effect of the miR-27a-3p inhibitor on PMECs proliferation,migration and mortality. The levels of BMPR2 were upregulated in circGSAP-overexpressed PMECs and lung tissues of MCT-PH rats.

**Conclusion:**

Our findings demonstrated that circGSAP alleviated the dysfunction of PMECs via the increase of BMPR2 by competitively binding with miR-27a-3p, and mitigated pulmonary vascular remodeling of MCT-PH rats, providing potential therapeutic strategies for IPAH.

**Supplementary Information:**

The online version contains supplementary material available at 10.1186/s12931-022-02248-7.

## Introduction

Idiopathic pulmonary arterial hypertension (IPAH) is characterized by the remodeling and obliteration of the pulmonary vasculature, resulting in a progressive increase in pulmonary vascular resistance and right heart pressure overload [1]. The dysfunction of endothelial cells (ECs) is regarded as the trigger of pulmonary vascular remodeling. ECs dysfunction may lead to neointima formation, inflammatory infiltration, fragmentation of the elastic lamina and vasoconstriction accompanied by muscularization and calcium deposits in the pulmonary arteries [2–5]. Thus, there is a need to identify the important molecules triggering ECs dysfunction and driving vascular remodeling, and to explore their therapeutic potential.

Dysregulated circular RNAs (circRNAs) play a role in the progression of pulmonary hypertension (PH) pathobiology [6–8], particularly in pulmonary vascular remodeling. Emerging in vivo studies have provided compelling evidence of the contribution of circRNAs to pulmonary vascular remodeling and PH pathogenesis. As such, several circRNA–microRNA (miRNA)–messenger RNA (mRNA) pathways have been identified as novel mechanisms of pulmonary vascular remodeling. Pulmonary arterial intimal injury is a well-defined feature of vascular remodeling in PH. There is ample evidence of endothelial dysfunction in PH, such as impaired angiogenic responses, metabolic changed and inappropriate activation of inflammatory cascades [2]. Although many postulate that the endothelium is the site of inciting injury and apoptosis followed by hyperproliferation and increased migration [9], the mechanisms by which circRNAs affect the functions of pulmonary microvascular endothelial cells (PMECs) in PH are not yet fully understood.

We recently found that low circRNA-gamma-secretase-activating protein (circGSAP) levels in peripheral blood mononuclear cells (PBMCs) were associated with IPAH occurrence and poor outcomes, indicating that circGSAP could be a biomarker for IPAH diagnosis and prognosis [10]. We also observed that diminished circGSAP facilitated cell proliferation and apoptosis resistance through competitive binding of miR-942-5p to modulate SMAD4 expressions in PMECs [11]. However, it is unclear whether circGSAP adsorbs other miRNAs to play a role in PEMCs dysfunction and experimental PH. Therefore, we aimed to investigate the underlying mechanisms by which circGSAP absorbs other miRNAs via the regulation of target genes to protect PMECs from dysfunction, which could yield novel therapeutic targets for IPAH.

## Methods

### Clinical samples

Human plasma samples from 41 incident patients with IPAH and 50 healthy individuals were obtained from the Shanghai Pulmonary Hospital, School of Medicine, Tongji University, from January 2015 to October 2022. The PH diagnosis was established according to the European Society of Cardiology and European Respiratory Society Guidelines [12]. Patients with other PH classifications or with diabetes were excluded from the study. This study was approved and supervised by the Ethics Committee of Shanghai Pulmonary Hospital (number: K20-150Y). Written informed consent was obtained from all participants.

### Cell lines

All primary human PMEC lines were purchased from ScienceCell (BK-3000, Shanghai, China). Briefly, primary PMECs were cultured in endothelial cell medium (BK-1001, Shanghai, China) supplemented with 10% fetal bovine serum (BK-1001, Shanghai, China), 1% penicillin/streptomycin (BK-1001, Shanghai, China), and 1% endothelial cell growth supplement (BK-1001, Shanghai, China) in a CO_2_ (5%) atmosphere at 37 °C. For cell culture under hypoxia, PMECs were exposed to CO_2_ (5%)/O_2_ (3%)/balance of N_2_ for 24 h.

### Plasmid, siRNA and cell transfection

The circGSAP sequence was amplified and cloned into the circRNA overexpression vector pLC5-ciR (Geneseed, Guangzhou, China). The primers used for cloning were shown in Additional file 1: Table S1. Three siRNAs targeting circGSAP and BMPR2 were designed and synthesized by GenePharma (Shanghai, China). The miR-27a-3p inhibitor, miR-27a-3p mimic, and negative control miRNA (NC) were synthesized by GenePharma (Shanghai, China). The sequences were shown in Additional file 1: Table S1. PMECs were transfected with the overexpression plasmid or siRNA by Lipo2000 Transfection Reagent (Invitrogen, USA). Briefly, the cells were seeded in 6-well plates, cultured for 24 h, and transfected with circGSAP siRNA, BMPR2 siRNA, and siRNA-NC using the Lipo2000 at a final concentration of 100 nM. PMECs at a confluence of 70–80% were transfected with 10 µL of siRNA and 10 µL of the Lipofectamine 2000 siRNA Transfection Reagent, which were separately diluted in 100 µL of DMEM. The reagents were incubated separately for 5 min, then mixed and incubated at room temperature for 15 min. The siRNA-transfection reagent mixture was added directly to the cells. Cells were placed in anoxic chamber 6 h later, and then cultured for another 24 h and used in subsequent experiments.

### Construction of the AAV-circGSAP and intratracheal injection

The circGSAP sequence was cloned into the pHBAAV-CMV-circ-EF1-ZsGreen vector, which was then packaged into AAVs from Hanheng Company (Hanheng Biotechnology Co., Ltd., Shanghai, China). Rats were anesthetized using isoflurane and administered 200 µL pHBAAV6-CMV-circGSAP-EF1-ZsGreen (AAV-circGSAP) (1.1 × 10^12^ vg/mL) via intratracheal injection. Control rats were treated with the control AAV. All experimental procedures were performed in accordance with and were approved by the Institute for Laboratory Animal Research at the Experimental Animal Centre of Tongji University, Shanghai, China.

### Animal studies

Sixty-eight male Sprague–Dawley (SD) rats weighing 180–220 g, aged 6–8 weeks, were obtained from Tongji University and maintained in a specific-pathogen-free environment. The experiments conformed to the Guide for the Care and Use of Laboratory Animals of the Institute for Laboratory Animal Research at the Experimental Animal Centre of Tongji University, Shanghai, China.

MCT-induced PH rats received a single intraperitoneal MCT injection (60 mg/kg body weight). Control rats received an equivalent volume of saline. After 30 days, the pulmonary vascular remodeling of the MCT-injected and control rats was assessed, and tissue samples were collected for subsequent experiments. All experimental procedures were performed in accordance with and were approved by the Institutional Animal Care and Use Committee of Tongji University.

### RNase R treatment

Two micrograms of total RNA were incubated for 20 min at 37 °C with or without 5 U/µg RNase R (Epicenter Technologies), subsequently purified by the RNeasy MinElute Cleaning Kit (Qiagen), and then analyzed by Quantitative Reverse transcription and real–time PCR (qRT–PCR).

### Cell transfection

For miR-27a-3p overexpression and inhibition, PMECs were transfected with 40 nM miRNA mimics (miR-27a-3p) or 80 nM miRNA inhibitor (anti-miR-27a-3p) (GenePharma, Shanghai, China) by using Lipo2000 Transfection Reagent (Invitrogen). The sequences were listed in Additional file 1: Table S1. The control groups were treated with equal concentrations of nontargeting mimic or inhibitor negative control sequences to eliminate nonspecific effects. The cells were seeded in 6-well plates, cultured for 24 h, and transfected with Lipo2000 (Invitrogen, CA) following the manufacturer’s protocol.

### Cell proliferation assay

Cell Counting Kit-8 (CCK-8, Dojindo, Japan) was used to measure cell proliferation. Briefly, the PMECs were seeded into 96-well plates at a density of 1.5 × 10^4^ cells per well and collected at different time points after the cells treated with 10 µL CCK-8 solution at 37 ℃ for 60 min. The absorbance at 450 nm was then detected, with a relative proliferation level obtained by normalization from at least three independent experiments.

### Wound healing assay

PMECs (70 µl) were seeded at a density of 1 × 10^6^ cells/mL into each Culture Insert-2 Well (Ibidi, cat. no. 81176). After 24 h of incubation, cell growth was arrested overnight by the addition of starvation medium (ECM containing 1% FBS). After the gentle removal of Culture-Insert 2 Well, the cells were washed with PBS to remove non-adherent cells. The images at 0 and 24 h were documented under microscope. The width of each scratch was measured using ImageJ software, and the migration ability of cells in each group was evaluated.

### Transwell assay with crystal violet staining

In brief, 200 µL cells (2 × 10^5^ cells) resuspended in free medium were added to the upper chamber (BD Biosciences). The bottom chamber was filled with 500 µL ECM with 5% FBS as an attractant. After 24 h, the cells that did not migrate were carefully swabbed out of the chamber. Then the cells were fixed with 4% paraformaldehyde solution and stained with 0.5% crystal violet and counted under a microscope.

### Calcein-AM/PI staining

The cells were analyzed for fluorescence by calcein-AM/PI staining (Calcein-AM/PI Double Stain Kit, Shanghai Yisheng Bio-Technology Co.,Ltd.). Living cells were visualized by calcein-AM (green fluorescence), while PI is used to stain dead cells (red channel). Briefly, PMECs were seeded in 24-well plates (2 × 10^5^ cells) for 24 h with or without treatment. The medium was discarded, and cells were washed with PBS for three times, followed by the incubation with a dyeing working fluid consisting of 2 µM calcein-AM and 2 µM PI for 30 min at 37 °C in the dark. Finally, the cells were analyzed using a fluorescence microscope.

### Dual-luciferase activity reporter system

Dual-luciferase reporter assays were performed to verify whether hsa_circGSAP or BMPR2 was the target of hsa-miR-27a-3p. hsa_circGSAP/BMPR2 wild type and mutant sequences were cloned downstream of the firefly luciferase gene pGL3 vector (Promega, USA). miR-27a-3p mimics were transiently co-transfected with luciferase-reporter plasmids (Promega) using Lipofectamine 2000 according to the manufacturer’s instructions.

pGL3-has_circGSAP vectors were co-transfected with miR-27a-3p mimics or negative control into 293T cells. After 48 h, firefly luciferase activity was measured using a dual-luciferase assay kit (Promega, Madison, USA) and normalized to Renilla luciferase activity. Each assay was repeated for a total of 5 independent experiments. The primer and oligonucleotide sequences were listed in Additional file 1: Table S1.

## ELISA assay

BMPR2 levels of PMECs under circGSAP regulation were determined using Human BMPR2 (Bone morphogenetic protein receptor type-2) ELISA Kit (EH4805, Wuhan Fine Biotech Co.) following the manufacturer’s protocol. Briefly, after washing PMECs with ice-cold PBS for three times, RIPA (P0013B, Beyotime Technology) was used to extract the proteins. The proteins were harvested after centrifuging at 15,000*g* for 30 min at 4 °C. Bicinchoninic acid protein (BCA) assay (23250, Pierce Chemical United States) was used to determine the protein concentrations. All the proteins were adjusted to the same concentration before the ELISA assay.

### Right ventricular systolic pressure (RVSP) and Fulton index measurement

After the SD rats were anesthetized with continuous isoflurane inhalation (1–2.5%), the right external jugular vein was exposed, and a polyethylene catheter with an internal diameter of 0.9 mm was inserted into the right ventricle (RV). The catheter was connected to a pressure recorder (BL-420 F) to record the RVSP. Then, SD rats were sacrificed, and heart and lung tissues were removed for subsequent experiments. The Fulton Index was calculated as RV/(LV + S) [the ratio of RV weight to left ventricle plus septum (LV + S) weight]. In addition, the left lung tissues were immersed in 4% paraformaldehyde, and the right lung tissues were stored at − 80 °C.

### Echocardiography and assessment of PH

Transthoracic echocardiography was performed with a Visual Sonics Vevo 2100 ultrasound machine. For anesthesia, rats were placed on a heated pad with continuous isoflurane inhalation (1–2.5%). The fur on the chest was removed with a chemical hair remover. Transthoracic echocardiography was performed to measure right ventricular end diastolic diameter (RVEDD) and pulmonary artery acceleration time (PAT).

### Histological examination

After euthanasia, the trachea of rats was separated. Next, the left lung lobe of mice was ligated, and the right lung was fixed by perfusion with 4% paraformaldehyde, followed by an additional regimen of fixation with 4% paraformaldehyde for 48 h for histological examination. Paraffin-fixed lung tissues were cut into 4 µM sections using a rotary microtome (Leica, Mannheim, Germany) and were then subjected to hematoxylin and eosin (HE) staining (Solarbio, Beijing, China). For immunohistochemistry staining, slides were stained with antibodies against ɑ-SMA (ab124964, Abcam, Cambridge, USA; 1:100). The orientation of collagen fibers was examined with Masson trichrome staining according to the manufacturer’s instruction. Then, these sections were microphotographed under a light microscope (DP73; Olympus Corporation, Tokyo, Japan).

### RNA fluorescence in situ hybridization (FISH)

The cell slides were fixed in 4% paraformaldehyde for 20 min and washed three times in phosphate buffered solution (PBS). The cells were digested by added proteinase K (20 µg/mL) for 5 min and washed with PBS for three times. The slides were treated with prehybridization solution for 1 h at 37 °C. Subsequently, the hybridization solution containing the fluorescently labeled junction probe at the concentration of 1 µM was added. The cells were hybridized at 42 °C overnight, followed by three washes with 1 × SSC for 10 min at room temperature. Images were captured using confocal microscopy. The sequence of the detection probe was listed in Additional file 1: Table S1.

### RNA and gDNA extraction

Total RNA was extracted from cells using TRIzol reagent (Invitrogen) according to the manufacturer’s instructions. The nuclear and cytoplasmic fractions were extracted using the PARIS Kit (Ambion, Life Technologies). Genomic DNA (gDNA) was extracted using the Genomic DNA Isolation Kit (Sangon Biotech, Shanghai, China).

### Quantitative reverse transcription and real-time PCR

Total RNA was extracted with a PureLink RNA mini kit (Thermo Fisher Scientific). For circRNA and mRNA expression detection, complementary DNA (cDNA) transcription from RNA was performed using ReverTra Ace qPCR RT Master Mix (TOYOBO, Japan). For miRNA expression, the RNA was reversed transcribed into cDNA by miRNA 1st Strand cDNA Synthesis Kit (Vazyme, China). After reverse transcription, cDNA was harvested, and the resulting cDNA was then subjected to real-time PCR analysis with SYBR Select Master Mix (Thermo Fisher Scientific) on a StepOne Plus real-time PCR system (Applied Biosystems). For each sample, the expression of each target gene were normalized to that of GAPDH or U6. Relative expression of genes was measured by the 2^−△△Ct^ method. The key primers were listed in Additional file 1: Table S1.

### Statistical analysis

The data were presented as the mean ± SD in the table and mean ± SEM in the figures. As indicated, we employed Student’s two-tailed unpaired t test to determine the statistical significance of differences in the in vitro experiments. We used one-way analysis of variance to compare continuous values between three or more groups and the log-rank test or Gehan–Breslow–Wilcoxon test to determine significant differences in the survival data. A *p* value of < 0.05 indicated statistical significance. Data management and analyses were performed with SPSS (version 19.0, Chicago, IL, USA), GraphPad Prism 8.0 software (GraphPad Software, La Jolla, CA). Figdraw (www.figdraw.com) was used to plot the schematic diagram. Results were representative of at least three independent experiments.

## Results

### CircGSAP is downregulated in the plasma of patients with IPAH and is associated with occurrence and poor outcomes

**Fig. 1:**
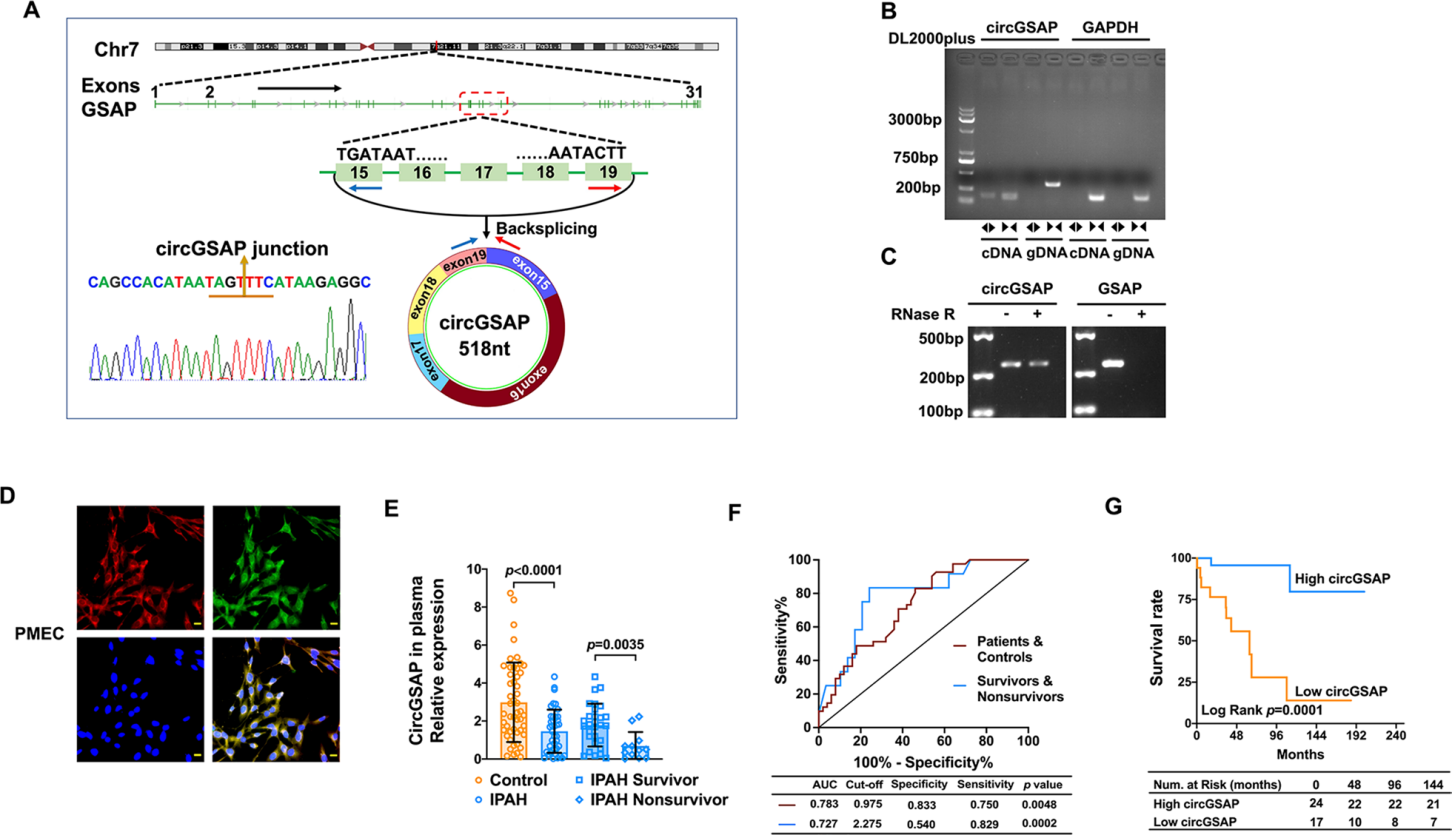
The characteristics, expression and impact of plasma circGSAP on IPAH. **A** Genomic loci of the circGSAP gene. CircGSAP is produced at the GSAP gene (NM_001350896.2) locus containing exons 15–19. The back-splice junction of circGSAP was identified by Sanger sequencing. **B** PCR analysis of circGSAP and linear GAPDH in cDNA and gDNA. **C** Validation of circGSAP by RNase R treatment and PCR analysis. **D** FISH was used to determine the distribution of circGSAP in PMECs. **E** Expression levels of circGSAP in plasma samples of IPAH, survivors and nonsurvivors were determined by qRT–PCR. **F** ROC curves of circGSAP in patients with IPAH, survivors and nonsurvivors with IPAH. **G** Kaplan–Meier survival analysis for mortality stratified by the cutoff values of circGSAP in IPAH. CircGSAP estimate of survival in patients with IPAH. Control n = 50, IPAH n = 41, survivors  n = 12, nonsurvivors n = 29. All data are presented as the mean ± SEM. Scale bar: 20 μm

Our previous study revealed that circGSAP was generated from GSAP exon 15 to exon 19 [10]. Sanger sequencing confirmed the junction sequence in the divergent primers spanning the predicted products (Fig. 1A). To further validate the construction of circGSAP, we designed convergent and divergent primers to amplify GSAP’s linear and circular products in cDNA and gDNA. Notably, circGSAP was amplified using divergent primers in cDNA samples, whereas no amplification product was observed in gDNA samples. The linear product of GSAP, however, was amplified in both cDNA and gDNA samples (Fig. 1B). To examine the resistance of circGSAP to RNase R digestion, we analyzed circGSAP and GSAP linear isoforms via reverse transcription (RT)-polymerase chain reaction (PCR) after RNase R treatment. The results showed that circGSAP was more resistant to RNase R treatment than GSAP linear isoform (Fig. 1 C). Previously, we also found that circGSAP was mostly located in the cytoplasm of PMECs [13] and circGSAP expression was decreased in hypoxia-induced PMECs [10], which were confirmed by fluorescent in situ hybridization (FISH) detection with a junction-specific circGSAP probe in this study (Fig. 1D).

We performed qRT–PCR analyses on plasma samples from 41 patients with IPAH and 50 healthy individuals. The baseline characteristics of the patients and healthy controls were shown in Table 1. The mean follow-up of patients was 67.5 ± 48.7 months. No patient was lost to follow-up. The expression of plasma circGSAP was significantly lower in groups of patients with IPAH versus the control groups and was lower in nonsurvivors than in survivors of IPAH patients (Fig. 1E), which was consistent with our previous finding of circGSAP in PBMCs and lung tissues of patients with IPAH [10]. Next, we constructed receiver operating characteristic (ROC) curves to assess the predictive capability of circGSAP. The area under the curve (AUC) for patients with IPAH was 0.727 (Fig. 1F), indicating that circGSAP might be a promising diagnostic indicator for IPAH. The AUC for circGSAP in patients who died from IPAH was 0.783 (Fig. 1F), indicating that circGSAP might also be a promising prognostic indicator for IPAH. Patients with IPAH with low plasma circGSAP levels had poorer outcomes (Fig. 1G).

**Table 1:** Baseline characteristics in patients with IPAH and healthy controls

Characteristic	IPAH patients (n = 41)	Control subjects (n = 50)
Age, years	46.1 ± 17.6	48.3 ± 14.3
Male/Female, n	14/27	20/30
Nonsurvivors, n	12	0
BMI, kg/m^2^	22.8 ± 4.4	–
NT-proBNP, pg/mL	797 (296–3035)	–
WHO-FC III/IV, n (%)	25 (59.2)	–
6MWD, m	362.2 ± 109.7	–
mRAP, mmHg	6.0 ± 5.1	–
mPAP, mmHg	56.2 ± 18.6	–
mPAWP, mmHg	8.3 ± 4.0	–
PVR, Wood units	15.8 ± 13.5	–
CO, L/min	3.5 ± 1.4	–
*Specific therapy, n (%)*		
PDE-5 inhibitors	10 (24.4)	–
ERAs	10 (24.4)	–
sGC stimulator	2 (4.9)	–
Combination	19 (46.3)	–

*6MWD* 6-minute walk distance, *BMI* body mass index, *CO* cardiac output, *ERA* endothelin receptor antagonist, *IPAH* idiopathic pulmonary arterial hypertension, *mPAP* mean pulmonary arterial pressure, *mPAWP* mean pulmonary capillary wedge pressure, *mRAP* mean right atrial pressure, *NT-proBNP* N-terminal pro-brain natriuretic peptide, *PDE-5* phosphodiesterase type 5, *PVR* pulmonary vascular resistance, *sGC* soluble guanylate cyclase, *WHO-FC* World Health Organization Functional Class

### CircGSAP alleviates the progression of MCT-induced PH in rats

**Fig. 2:**
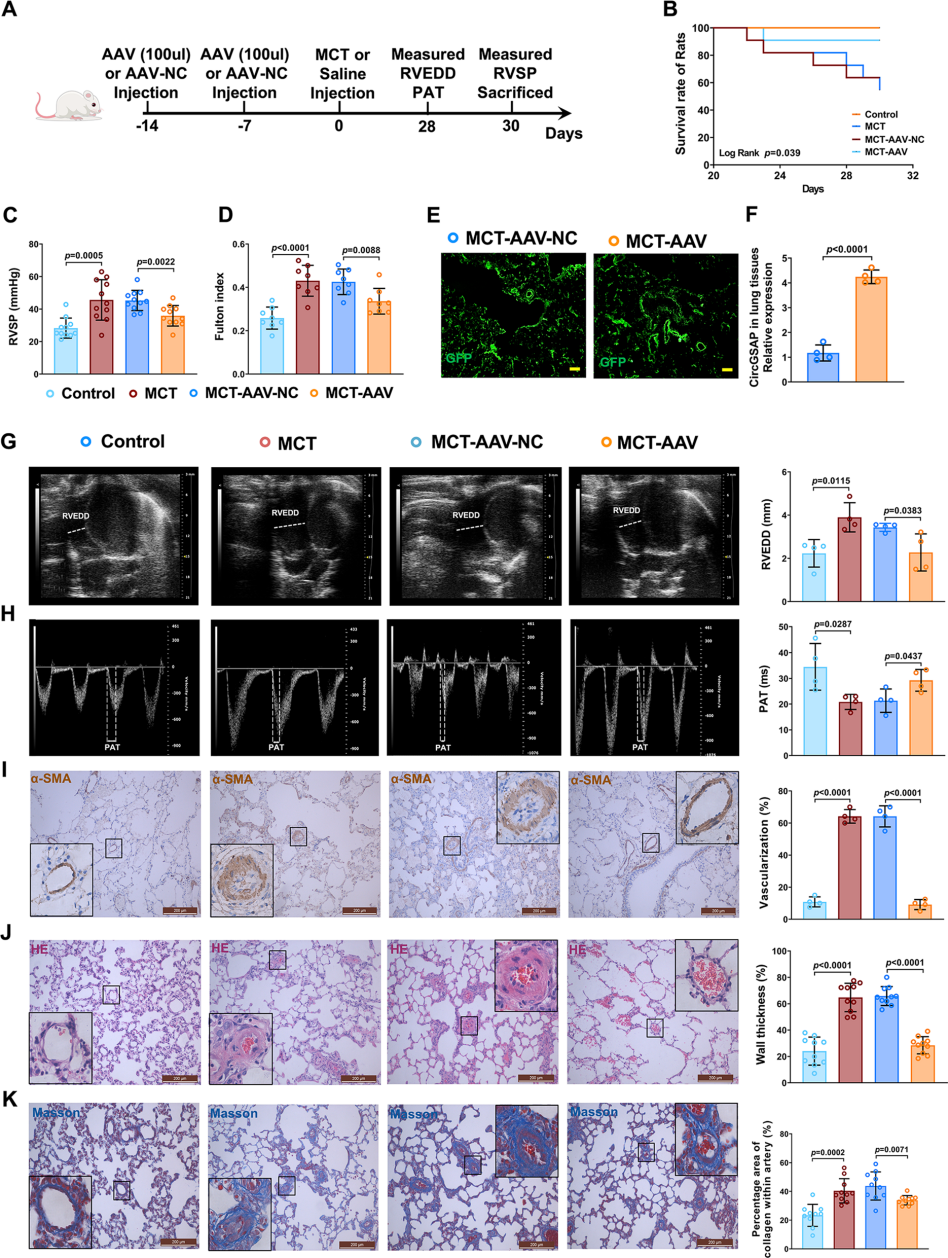
Effects of circGSAP on the progression of MCT-induced PH in rats. **A** Flow chart of the animal experiment. **B** Kaplan–Meier survival analysis for MCT-induced PH rats (n = 17). **C**, **D** RHC analysis of RVSP and the Fulton index in the control, MCT, MCT-AAV-NC and MCT-AAV groups (n = 11 or 8). **E**, **F** GFP fluorescent signals in the pulmonary arterial intima and the expression of circGSAP in MCT-AAV lung tissues (n = 4). **G**, **H** Echocardiography analysis of the RVEDD and PAT in the control, MCT, MCT-AAV-NC and MCT-AAV groups (n = 4). **I–K** Morphological analysis of the pulmonary artery was performed using α-SMA, HE and Masson staining (n = 4 or 10). All data are presented as the mean ± SEM. Scale bar: 100 μm

We next investigated whether circGSAP regulates pulmonary vascular remodeling in vivo. We constructed an adeno-associated virus (AAV) vector based on circGSAP (AAV-circGSAP) and delivered it intratracheally into rats before MCT injection (Fig. 2A). Stable transfection was observed, as evidenced by green fluorescent protein (GFP) signals in pulmonary arterial intima, and circGSAP expression was significantly increased in MCT-AAV lung tissues (Fig. 2E, F). AAV-circGSAP improved the survival and significantly attenuated the elevation of RVSP and the Fulton index in MCT-induced PH rats (Fig. 2B–D). Echocardiography showed that AAV-circGSAP significantly attenuated the RVEDD and extended the PAT (Fig. 2G, H). The number of fully muscularized vessels, wall thickness and collagen fiber accumulation were significantly reduced in the AAV-circGSAP group (Fig. 2I–K). These results showed that circGSAP alleviates the progression of MCT-induced PH in rats.

### CircGSAP inhibits PMECs overproliferation and migration and promotes its mortality

**Fig. 3:**
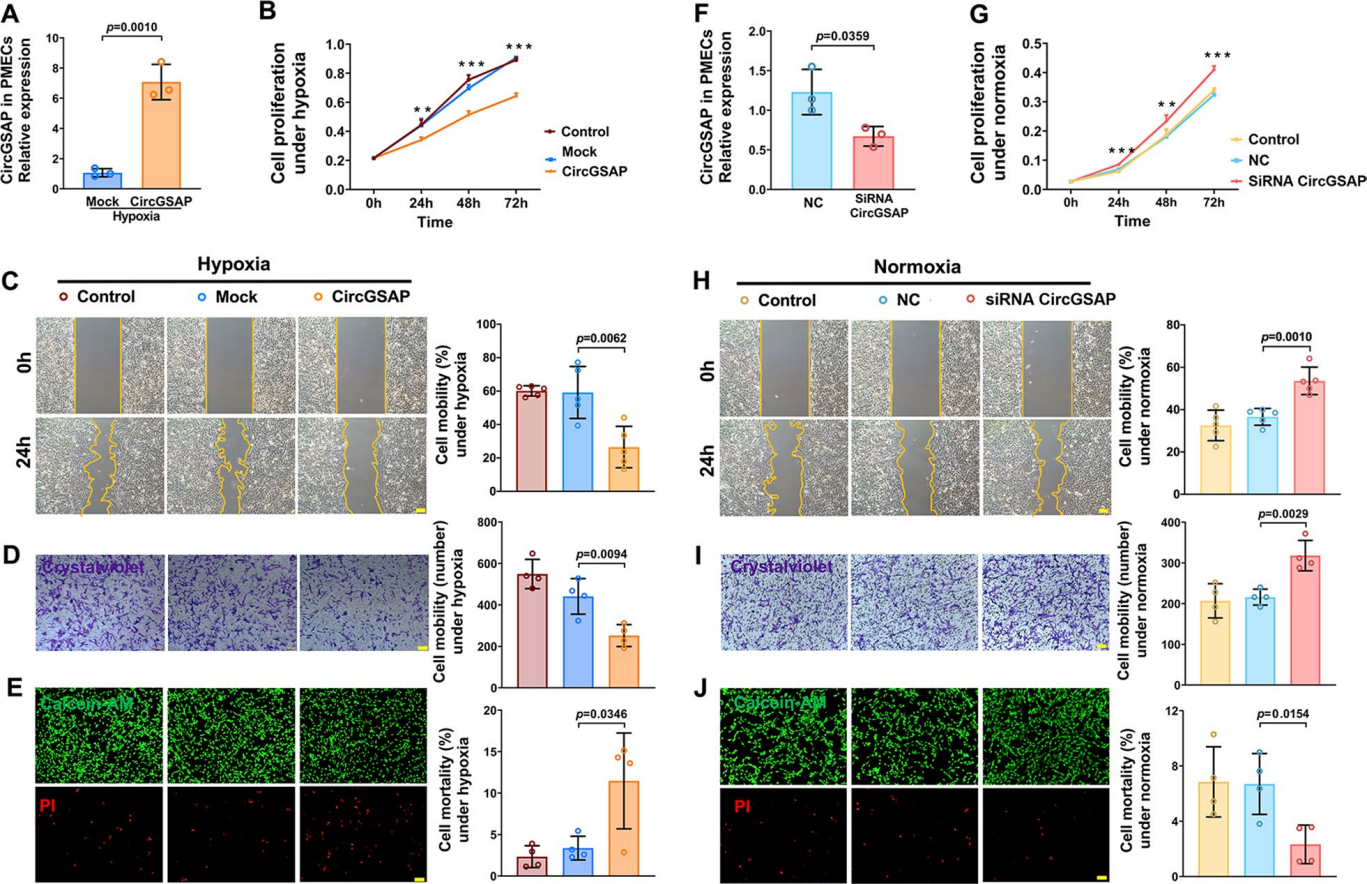
Effects of circGSAP on the proliferation, migration and mortality of PMECs. **A** Expression levels of circGSAP in PMECs treated with circGSAP under hypoxia (n = 3). **B–D** Cell proliferation, wound healing and migration analyses of PMECs overexpressing circGSAP under hypoxia (n = 5 or 4). **E** Cell mortality analysis of PMECs overexpressing circGSAP under hypoxia (n = 4). **F** Expression levels of circGSAP in PMECs after transduction with circGSAP siRNA (n = 3). **G–J** Cell proliferation analysis, wound healing analysis, cell migration analysis, and cell mortality analysis of PMECs with silencing circGSAP under normoxia (n = 5 or 4). All data are presented as the mean ± SEM. Scale bar 100 μm. *p < 0.05; **p < 0.01; ***p < 0.001

Given that circGSAP is downregulated in the lung tissue of patients with IPAH and hypoxia-induced PMECs, we overexpressed circGSAP to further study its potential function in PMECs under normoxic and hypoxic conditions. As shown in Fig. 3A, circGSAP was significantly overexpressed in PMECs via circGSAP plasmid transfection. The upregulation of circGSAP reduced cell proliferation under hypoxic conditions (Fig. 3B). Moreover, wound healing and transwell assays demonstrated that circGSAP overexpression significantly inhibited PMECs migration (Fig. 3C, D). We also investigated whether circGSAP affected cell mortality using an AM/PI Kit. The results showed that cell mortality could be increased by circGSAP overexpression (Fig. 3E). Under normoxic conditions, overexpression of circGSAP also inhibited the proliferation, migration and increased mortality of PMECs, as shown in Additional file 2: Fig. S1.

To further confirm the function of circGSAP, we designed siRNA targeting circGSAP that efficiently knocked down the expression of circGSAP in cells under normoxic conditions (Fig. 3F). It was shown that circGSAP depletion increased PMECs proliferation according to CCK-8 assay (Fig. 3G). Furthermore, circGSAP silencing significantly increased PMECs migration (Fig. 3H, I). The results of the AM/PI Kit also showed that circGSAP silencing reduced mortality (Fig. 3J). These results suggest that circGSAP plays a vital role in regulating PMECs function.

### CircGSAP interacts with miR-27a-3p in PMECs

**Fig. 4:**
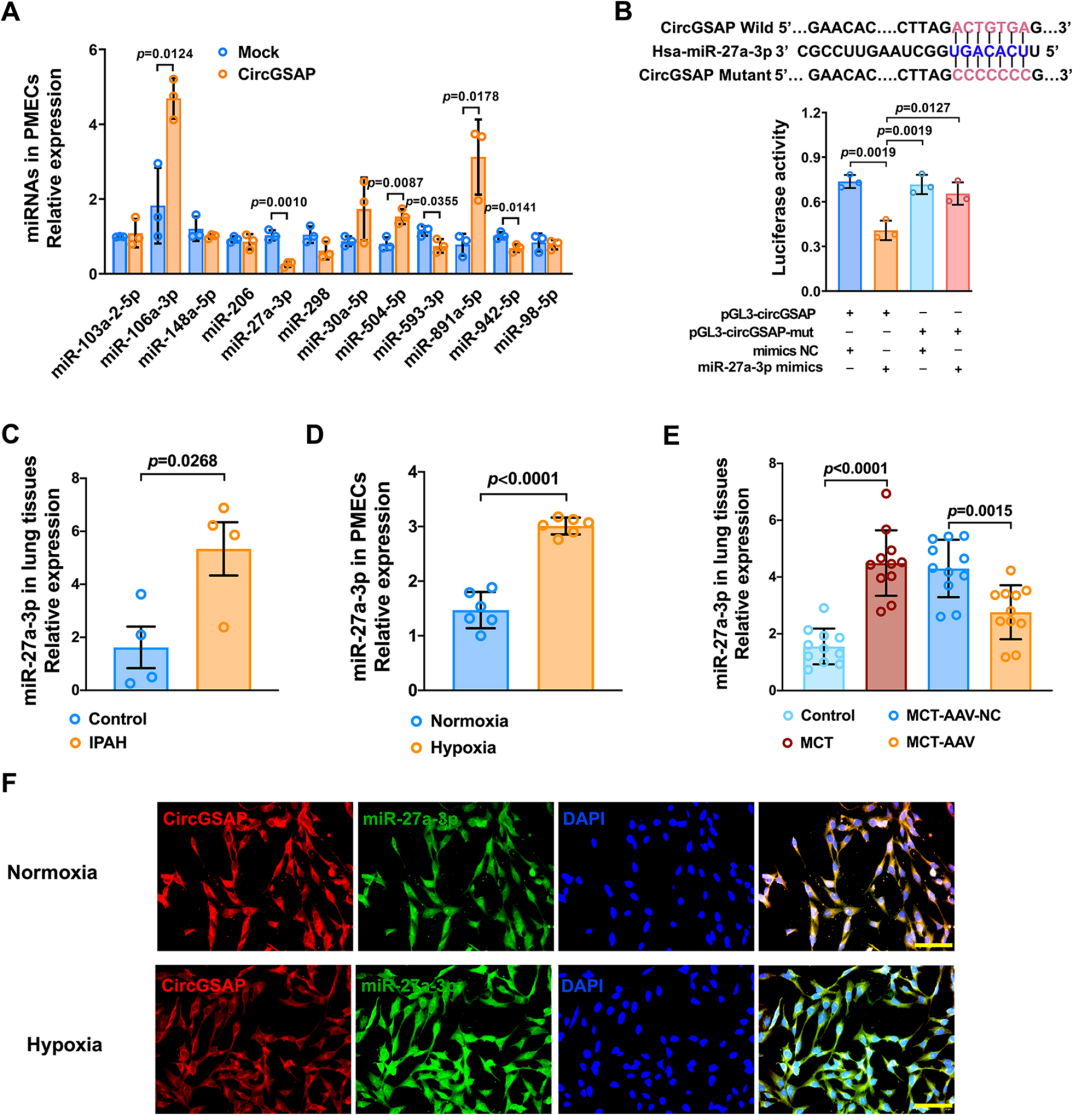
The interaction between circGSAP and miR-27a-3p in PMECs. **A** qRT–PCR was performed to detect miRNA expression levels associated with circGSAP (n = 3). **B** TargetScan predicted the binding sites of circGSAP and miR-27a-3p, and dual-luciferase assays were used to validate the interactions between circGSAP and miR-27a-3p (n = 3). **C** Expression levels of miR-27a-3p in lung tissues of IPAH patients were determined by qRT–PCR (n = 4). **D** Expression levels of miR-27a-3p PMECs were determined by qRT–PCR (n = 6). **E** Expression levels of miR-27a-3p in lung tissues of control, MCT, MCT-AAV-NC and MCT-AAV group rats (n = 11). **F** FISH was performed to observe the colocalization of circGSAP and miR-27a-3p in the cytoplasm of PMECs. All data are presented as the mean ± SEM. Scale bar: 100 μm

In terms of the molecular mechanism of circGSAP in IPAH, we have previously found that circGSAP was mainly expressed in the cytoplasm of PMECs [13]. Other studies have demonstrated that some circRNAs expressed in the cytoplasm can function as miRNA sponges in PH [7, 14, 15]; thus, we combined several bioinformatics websites (Circular RNA Interactome, circBANK, circAtlas 2.0) to predict miRNAs that potentially bind to circGSAP. Twelve miRNAs were selected and verified using qRT–PCR, and we found that miR-27a-3p, miR-593-3p and miR-942-5p could bind to circGSAP (Fig. 4A). We next manually selected miR-27a-3p for experimental verification. Dual-luciferase reporter assays were performed with a recombinant reporter plasmid containing a luciferase gene and the circGSAP sequence (pGL3-circGSAP). A schematic of the pGL3-circGSAP and circGSAP recognition sites was shown in Fig. 4B. Cotransfection of pGL3-circGSAP and miR-27a-3p significantly reduced firefly luciferase reporter activity (Fig. 4B), indicating that miR-27a-3p can bind to circGSAP. Furthermore, the relative expression of miR-27a-3p was significantly increased in IPAH patients and PMECs under hypoxic conditions (Fig. 4C, D). The expression of miR-27a-3p was also upregulated in the lung tissues of MCT-induced PH rats, but remarkably reduced in MCT-AAV lung tissues (Fig. 4E). Finally, FISH assays indicated that miR-27a-3p and circGSAP colocalized in PMECs (Fig. 4F).

### The miR-27a-3p inhibitor inhibits PMECs overproliferation and migration and promotes its mortality

**Fig. 5:**
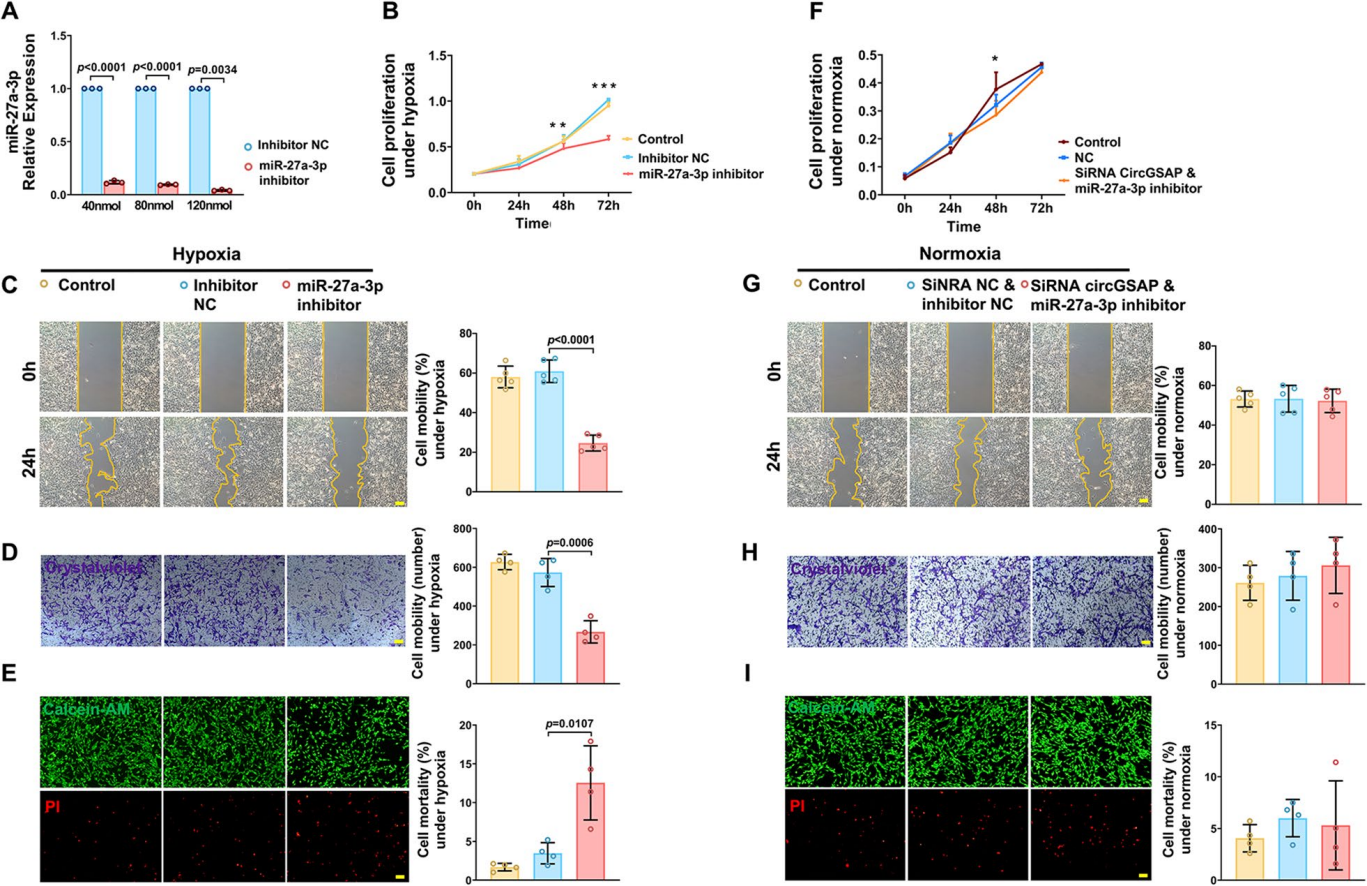
Effects of miR-27a-3p on the proliferation, migration and mortality of PMECs. **A** Expression levels of miR-27a-3p in PMECs treated with miR-27a-3p inhibitor (n = 3). **B–E** Cell proliferation analysis, wound healing analysis, cell migration analysis and cell mortality analysis of PMECs with miR-27a-3p inhibitor under hypoxia (n = 5 or 4). **F–I** Cell proliferation analysis, wound healing analysis, cell migration analysis and cell mortality analysis of PMECs cotransfected with circGSAP and miR-27a-3p inhibitor under normoxia (n = 5 or 4). All data are presented as the mean ± SEM. Scale bar 100 μm. *p < 0.05; **p < 0.01; ***p < 0.001

To examine the effects of miR-27a-3p on PMECs proliferation, migration and mortality, we first used a miR-27a-3p inhibitor or its negative control (NC) to transfect PMECs. The knockdown efficiency was validated using qRT–PCR 24 h later. A significant downregulation of miR-27a-3p expression was observed in the miR-27a-3p inhibitor group compared with the NC group (Fig. 5 A). The results showed that miR-27a-3p knockdown inhibited proliferation and migration and increased mortality in cultured PMECs under hypoxic conditions (Fig. 5B–E). Similar results were observed under normoxic conditions, as shown in Additional file 3: Fig. S2. After cotransfection of PMECs with the miR-27a-3p inhibitor and siRNA circGSAP, we found that inhibiting the expression of miR-27a-3p significantly reversed the PMECs overproliferation, migration and mortality reduction caused by circGSAP silencing under normoxic conditions (Fig. 5F–I). These results further confirmed that circGSAP can competitively combine with miR-27a-3p in PMECs.

### BMPR2 is the target of miR-27a-3p in PMECs

**Fig. 6:**
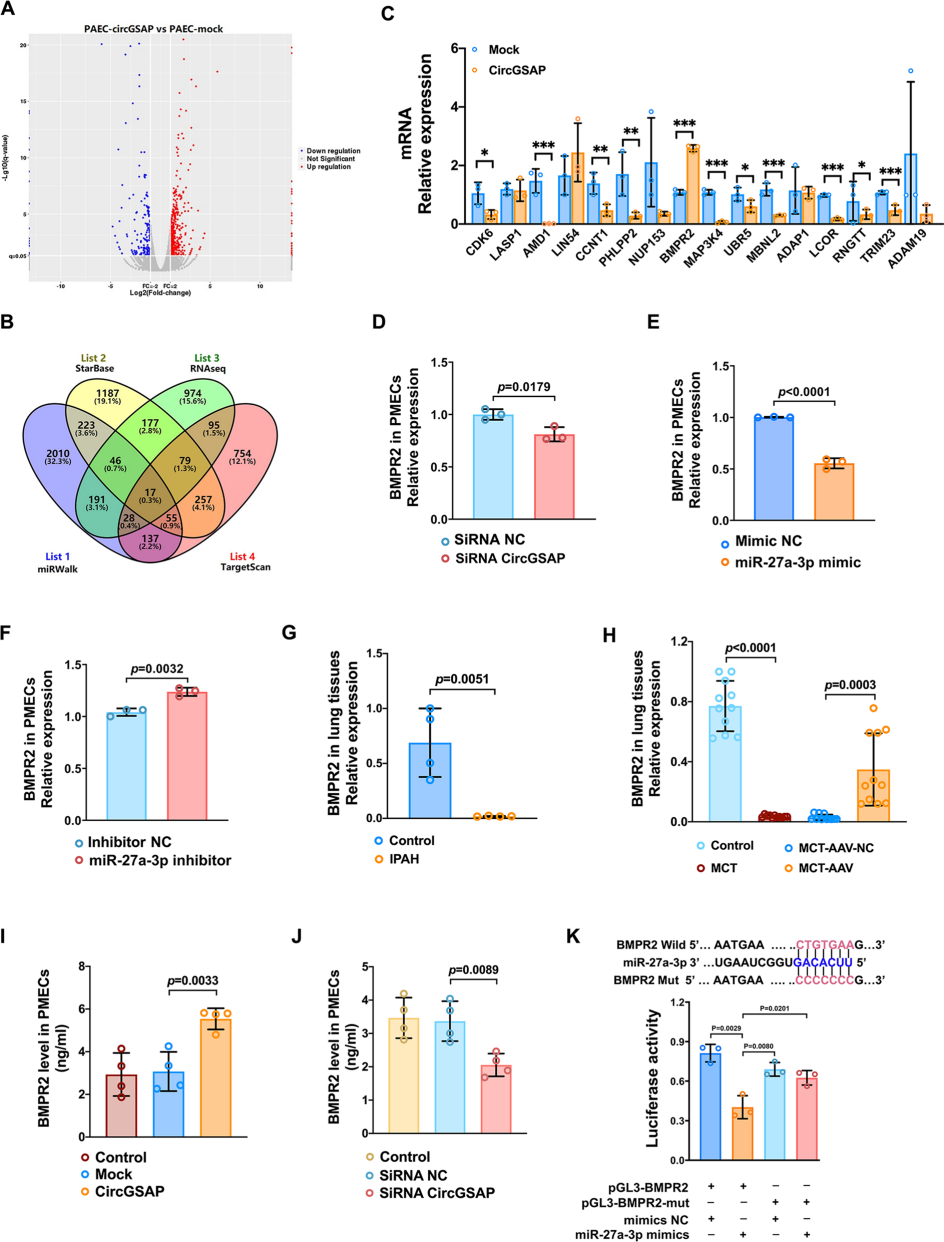
BMPR2 is the target of miR-27a-3p. **A** Volcano map analysis of RNA sequencing in PAECs overexpressing circGSAP. **B** miRWalk, StarBase, and TargetScan bioinformatics software predicted mRNAs that could bind to miR-27a-3p and the intersection with RNA sequencing results. **C** qRT–PCR was used to detect mRNA that could bind to miR-27a-3p in PMECs treated with circGSAP (n = 3). **D** Expression levels of *BMPR2* in PMECs treated with circGSAP siRNA (n = 3). **E**, **F** Expression levels of *BMPR2* in PMECs treated with miR-27a-3p mimics and inhibitor (n = 3). **G** Expression levels of *BMPR2* in lung tissues of IPAH patients (n = 4). **H** Expression levels of *BMPR2* in lung tissues of control, MCT, MCT-AAV-NC and MCT-AAV group rats (n = 11). **I**, **J** The protein levels of BMPR2 in circGSAP-treated and siRNA circGSAP-treated PMECs (n = 4). **K** Dual-luciferase assays were used to validate the interactions between miR-27a-3p and *BMPR2 * (n = 3). All data are presented as the mean ± SEM. Scale bar: 100 μm

In this study, we found that 17 genes could be potential miR-27a-3p targets (Fig. 6A, B) according to RNA sequencing analysis of circGSAP overexpressed PMECs versus control PMECs with three bioinformatics algorithms (miRWalK, StarBase and TargetScan). We subsequently performed qRT–PCR to validate 17 genes and found that *BMPR2*, a central key mediator not only of development but also of vascular homeostasis in IPAH, was upregulated in circGSAP-treated PMECs (Fig. 6C) and was downregulated in circGSAP silencing PMECs (Fig. 6D). qRT–PCR showed that *BMPR2* expression was negatively regulated by miR-27a-3p (Fig. 6E, F). The expression of *BMPR2* was also downregulated in the lung tissues of IPAH patients and MCT-induced PH rats, but significantly upregulated in MCT-AAV lung tissues (Fig. 6G, H). The protein levels of BMPR2 were also upregulated in circGSAP-overexpressed PMECs, while that were downregulated in circGSAP silencing PMECs (Fig. 6I, J). Results from the dual-luciferase reporter assay showed that the luciferase activity of the wild-type, but not the mutant, BMPR2-3′ UTR reporter was significantly decreased in PMECs transfected with miR-27a-3p mimics (Fig. 6K).

### Silencing BMPR2 promotes PMECs overproliferation and migration and reduces PMECs mortality

**Fig. 7:**
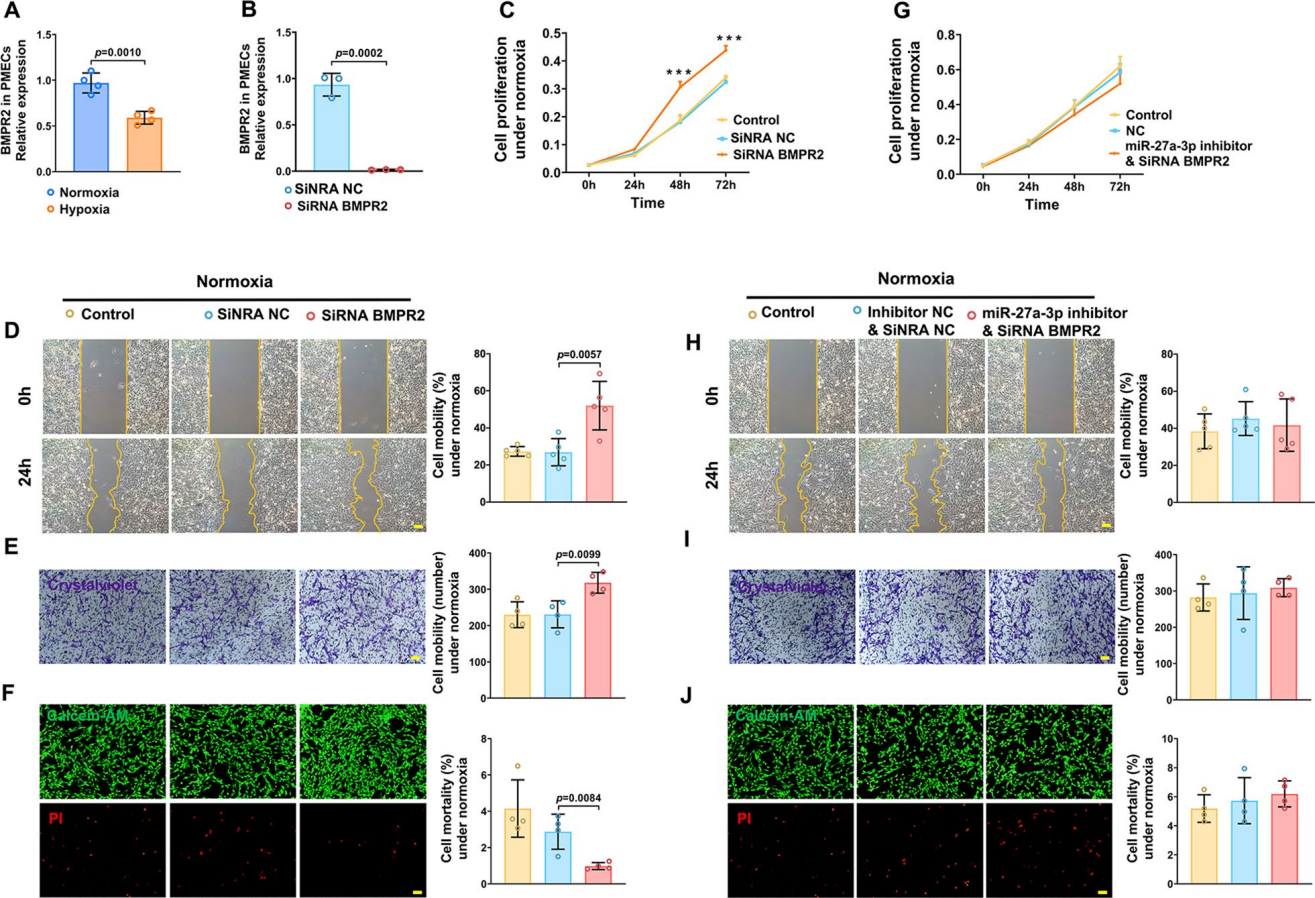
Effects of *BMPR2* on the proliferation, migration and mortality of PMECs. **A** Expression levels of BMPR2 in hypoxia-treated PMECs (n = 4). **B** Expression levels of BMPR2 in PMECs treated with siRNA BMPR2 (n = 3). **C–F** Cell proliferation analysis, wound healing analysis, cell migration analysis and cell mortality analysis of PMECs transfected with siRNA BMPR2 under normoxia (n = 5 or 4). **G–J** Cell proliferation analysis, wound healing analysis, cell migration analysis and cell mortality analysis of PMECs cotransfected with miR-27a-3p inhibitor and siRNA BMPR2 under normoxia (n = 5 or 4). All data are presented as the mean ± SEM. Scale bar: 100 μm

*BMPR2* expression was decreased in hypoxia-treated PMECs (Fig. 7A). When PMECs were transfected with siRNA targeting BMPR2, *BMPR2* expression was significantly knocked down in PMECs (Fig. 7B). We next observed that BMPR2 knockdown promoted PMECs proliferation and migration and reduced PMECs mortality under normoxic conditions (Fig. 7C–F). More importantly, a miR-27a-3p inhibitor and siRNA BMPR2 were cotransfected into PMECs; the effect of the miR-27a-3p inhibitor on proliferation, migration and mortality was reversed in *BMPR2* siRNA-treated PMECs (Fig. 7G–J), suggesting that miR-27a-3p affected the roles of BMPR2 in PMECs.

## Discussion

**Fig. 8:**
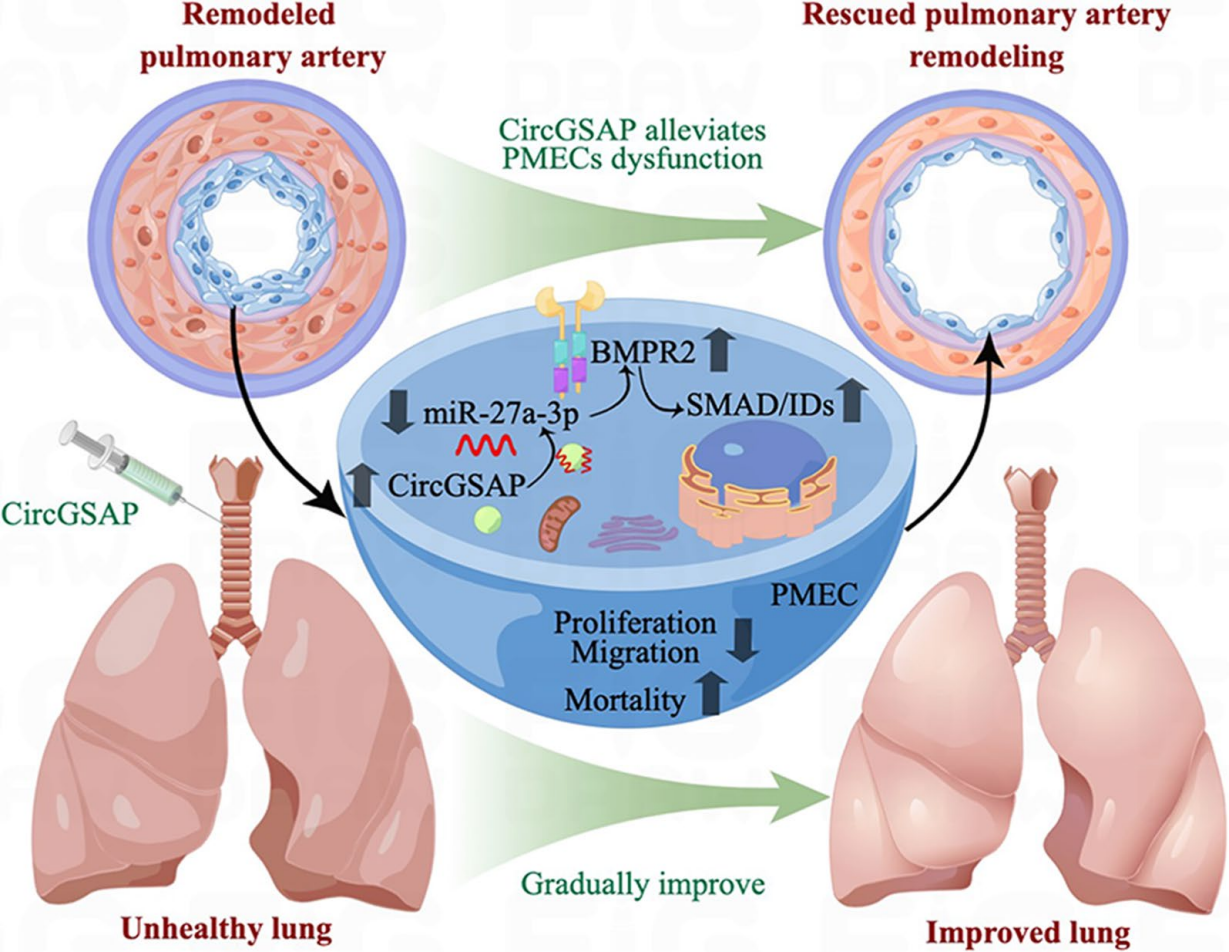
A schematic diagram illustrating the hypothetical model by which circGSAP adsorbed miR-27a-3p via a sponging mechanism and increased the BMPR2 signaling pathway, improving the overproliferation and migration, increasing mortality of PMECs in the remodeled pulmonary arteries

Multiple lines of evidence have shown that circRNAs play roles in the development and progression of PH [7, 10, 14, 16, 17]. In a previous study, we confirmed that lower circGSAP levels in PBMCs could be an emerging biomarker for IPAH diagnosis and prognosis evaluation [10]. Here, we found that downregulation of plasma circGSAP was associated with IPAH development and poorer outcomes and that overexpression of circGSAP improved the progression of MCT-induced PH in rats. The pulmonary arterial intima was found to be involved in the pathogenesis of IPAH. We then investigated the potential regulatory effects of circGSAP on PMECs function. Our results revealed that circGSAP could bind to miR-27a-3p and markedly decreased its activity. Therefore, we proposed the following mechanism: circGSAP acts as a sponge to suppress the functions of miR-27a-3p and increase *BMPR2* levels, leading to the inhibition of PMECs proliferation and migration, increase of mortality, thus alleviating pulmonary arterial intimal injury in IPAH (Fig. 8).

CircRNAs are a type of widespread and conserved endogenous noncoding RNA in mammalian cells. Many studies have indicated that specific circRNAs could be emerging biomarkers for the diagnosis and prognosis of various diseases [18–20]. In our previous study, we observed that lower circGSAP levels in PBMCs were associated with the occurrence of IPAH and poor outcomes [10, 21]. According to these data, we enrolled patients with IPAH to examine the plasma level of circGSAP and its role in prediction of IPAH occurrence and prognosis in these patients. We further confirmed that low circGSAP levels could serve as a reliable predictive biomarker in patients with IPAH. However, although circGSAP is expressed in patients’ PMECs, PBMCs, lung tissue and plasma, we are uncertain about the original source of circGSAP. Therefore, further investigation including single-cell sequencing and other technologies to address this issue is needed.

The endothelium is a cellular monolayer that covers the inner lining of the entire circulatory system. Multiple PH subtypes, including IPAH, are driven though PMECs dysfunction. Through its strategic position in the vascular wall, the endothelium responds to changes in blood oxygen and nutrient content by producing signals that affect vascular tone, barrier permeability and circulating cell recruitment. A well-known marker of endothelial dysfunction is reduced production of vasodilators (e.g., nitric oxide and prostaglandins) in lieu of vasoconstrictive factors (e.g., thromboxane A2 and endothelin 1) [2]. The present study indicates that the downregulation of circGSAP promotes PMECs proliferation and migration and reduces PMECs mortality, leading to the progression of pulmonary arterial remodeling and right ventricular hypertrophy in MCT-induced PH rats. The overexpression of circGSAP was proved to alleviate pulmonary artery intimal injury and inhibit pulmonary vascular remodeling by regulating PMECs dysfunction in the pathogenesis of PH.

Although the specific functions of most circRNAs remain unclear, accumulating evidence had revealed a role of circRNAs as miRNA sponges [15, 22]. The lack of free ends allows circRNAs to evade the destabilization and degradation mediated by miRNAs. Several recent studies have indicated the availability of circRNAs as miRNA sponges to take part in pulmonary arterial remodeling related to PH by regulating the function of pulmonary arterial smooth muscle cells (PASMCs) [7, 14, 16, 17]. However, few studies have focused on the regulation of circRNAs as miRNA sponges in the dysfunction of PMECs during the progression of pulmonary arterial remodeling in PH. The current study demonstrates that decreased circGSAP might promote overproliferation and migration, and reduce mortality by sponging miR-27a-3p to regulate *BMPR2* expression in PMECs.

A series of past studies have detected miR-27a-3p expression in various diseases; for example, miR-27a-3p is downregulated in tumors and cardiovascular disease [13, 23] and upregulated in obesity and neurodegenerative diseases [24, 25]; however, there is a lack of research on its expression profile in PH. In our study, we found that miR-27a-3p expression was increased in hypoxia-exposed PMECs, and a miR-27a-3p inhibitor suppressed the proliferation and migration and increased the mortality of hypoxia-exposed PMECs. These data indicated that the upregulation of miR-27a-3p might be involved in PH pathogenesis. However, some studies have revealed a protective role of miR-27a-3p to inhibit smooth muscle cell proliferation in vitro [26, 27], which is inconsistent with our results. A possible reason could be that the studied cells and their cellular microenvironment were different.

BMPR2 deficiency has been inexorably related to pathologic pulmonary vascular changes, poorer clinical outcomes and increased susceptibility to IPAH [28, 29]. The loss of BMPR2 directly induced DNA damage and mitochondrial dysfunction and facilitated a transition from glucose oxidation to glycolysis [30], leading to a disrupted cytoskeletal and adhesion structure, inhibited apoptosis, increased proliferation and migration and the secretion of vasodilatory and inflammatory cytokines by endothelial cells [30, 31], all of which occur in PH. There are multiple reasons for the downregulation of the BMPR2 signaling pathway that do not involve genetic mutations and that could contribute to reducing BMPR2 below a critical threshold needed to initiate PH [32, 33]. A previous study demonstrated that the dysregulation of miR-20a and miR-130a/b could activate downstream BMPR2 to regulate PASMCs proliferation [34, 35]. Similarly, our results suggest that BMPR2 is a target of miR-27a-3p in PMECs and that overexpressed circGSAP could upregulate *BMPR2* by sponging miR-27a-3p in PMECs, and thus mitigating the progression of PH.

The present study has also several limitations. The question of whether circGSAP modulates TGF-β–Smad2/3 signaling as well remains unaddressed and needs to be explored in future studies, given a close link of TGF-β signaling to bone morphogenetic protein signaling, which could be regulated by circGSAP. Second, our results suggest that circGSAP can alleviate disease progression in MCT-PH rats. Given that the MCT model is an inflammatory model, the effect of circGSAP on proinflammatory cytokines in vitro and in vivo needs to be studied as well. Third, although we observed the effect of circGSAP on PMECs proliferation, migration and mortality via miR-27a-3p, it warrants further investigation whether circGSAP could play a protective role in the context of PH pathobiology via other possible mechanism such as binding RNA binding proteins and coding proteins or polypeptides. Additionally, from the localization data, we observed that circGSAP can be expressed in PASMCs or inflammatory cells around blood vessels. Whether circGSAP affects the function of PASMCs or participates in immune and inflammatory regulation in the pathogenesis of PH also needs further exploration to obtain a more comprehensive understanding of the underlying mechanism by which circGSAP alleviates the progression of PH.

## Conclusion

Low circGSAP levels are associated with poor outcomes in patients with IPAH, indicating that circGSAP could be an emerging biomarker for IPAH prognosis. Furthermore, downregulated circGSAP facilitates the dysfunction of PMECs by competitively binding with miR-27a-3p to decrease the level of BMPR2 in PMECs, which could provide opportunities for potential IPAH therapeutic strategies.

## Supplementary Information

**Additional file 1: Table S1.** The sequences of primers, siRNA and mimics or inhibitor used in this study.

**Additional file 2: Fig. S1.** Effects of circGSAP on the proliferation, migration and mortality of PMECs under normoxia. **A** Expression levels of circGSAP in PMECs treated with circGSAP under normoxia (n = 3). **B-D** Cell proliferation analysis, wound healing analysis and cell migration analysis of PMECs overexpressing circGSAP under normoxia (n = 5 or 4). **E** Cell mortality analysis of PMECs overexpressing circGSAP under normoxia (n = 4). All data are presented as the mean ± SEM. *p < 0.05; Scale bar 100 μm. **p < 0.01; ***p < 0.001.

**Additional file 3: Fig. S2.** Effects of miR-27a-3p on the proliferation, migration and mortality of PMECs under normoxia. **A-D** Cell proliferation analysis, wound healing analysis, cell migration analysis and cell mortality analysis of PMECs with miR-27a-3p inhibitor under normoxia (n = 5 or 4). All data are presented as the mean ± SEM. *p < 0.05; **p < 0.01; ***p < 0.001.

## Data Availability

All the relevant raw data and materials are freely available from the corresponding author on reasonable request.

## Abbreviations

ECs: Endothelial cells
IPAH: Idiopathic pulmonary arterial hypertension
MCT: Monocrotaline
PAT: Pulmonary artery acceleration time
PBMCs: Peripheral blood mononuclear cells
PH: Pulmonary hypertension
PMECs: Pulmonary microvascular endothelial cells
RVEDD: Right ventricular end diastolic diameter
RVSP: Right ventricular systolic pressure
