# Supplementary material for: CircGSAP alleviates pulmonary microvascular endothelial cells dysfunction in pulmonary hypertension via regulating miR-27a-3p/BMPR2 axis
Source: Respir Res. 2022 Nov 19;23:322. doi: 10.1186/s12931-022-02248-7 (PMC9675109; doi:10.1186/s12931-022-02248-7)
Supplement: Supplementary file 1 — Additional file 1: Table S1. The sequences of primers, siRNA and mimics or inhibitor used in this study. [file 12931_2022_2248_MOESM1_ESM.docx]

**Supplemental file 1**

| **Table S1. Primers, siRNA and mimics or inhibitors in study** | |
| --- | --- |
| **Primers** |  |
| circGSAP | F: ACAGAAGACATTGCATTGCCT |
|  | R: AGAGATTGTGGCAGATCAGGT |
| GSAP | F: GCTACCTCATTGCCCTTTACAG |
|  | R: ACTTGTCTCTGAATATACACTCCAG |
| GAPDH | F: TCTCTGCTCCTCCTGTTCGA |
|  | R: GCGCCCAATACGACCAAATC |
| miR-27a-3p | F: ACACTCCAGCTGGGTTCACAGTGGCTAAG |
|  | R: GTGCAGGGTCCGAGGT |
| U6 | F: TGCTTCGGCAGCACATATAC |
|  | R: TCACGAATTTGCGTGTCATC |
| BMPR2 | F: AGATCCTGGGCCATCAAAGC |
|  | R: TCACCTGATCCTGATTTGCCA |
| SMAD4 | F: GCCCAGGATCAGTAGGTGGA |
|  | R: GGTCCCCAGCCTTTCACAAA |
| ID3 | F: GCTCACTCCGGAACTTGTCA |
|  | R: TGGTGAAGTCAAGTGGGCAG |
| CDK6 | F：CCCCTCAGGTGCAATGATTC  R：ATCCCTCCTCTTCCCTCCTC |
| LASP1 | F：CAGCCCCAGTCTCCATACAG  R：CTCCACCGTCCCGTACATC |
| CCNT1 | F：ATTCACCTGGCTTGCAAGTG  R：TGTTCCTCGGTCATCTGCTT |
| NUP153 | F：AGAATCACACCTGAGCCAGC  R：GCTGACAGTGTAATGCAGGG |
| UBR5 | F：AGCGACTCTCCATGGTTTCT  R：CCCGTCCTGTCCGAATAACT |
| ADAP1 | F：CTGGTTCAATGCACTCCGAG  R：TGCCAATGAAGACTTCCCCT |
| RNGTT | F：TTCAATTCACAGCCCGTTGG  R：CATCACATCGACCAGGTTTGT |
| SPATA13 | F：GCCGAGCTGCTCAAGTATAC  R：GATATCCAGTCCCTCCCAGC |
| AMD1 | F：AGTCGGGTAATCAGTCAGCC  R：TCTGGTTCTGGAGTGATGTGA |
| LIN54 | F：GGGTTATGCAGTGCTTCCAG  R：GGCCGACTCTGATGGGATTA |
| PHLPP2 | F：GTACCGTGGATCTCTCGTGT  R：AGTTCAGGCCCTTCAGTTGA |
| MAP3K4 | F：ATCCAGTCAGCCGGTCATC  R：ACTCCCCAGACATCAAACGA |
| MBNL2 | F：ACCTGTAACCCCTGGAGTTG  R：GTGTCGATCATGGTGCTGTC |
| LCOR | F：GCTGACCAAGACTCACCTCT  R：CCGTTCCCTTGAGTACTGGA |
| TRIM23 | F：CGAGATGCTCTGCTCCTGAT  R：CTACAAGTTGCCGTGAGAGC |
| ADAM19 | F：CACTCCGAGAATGCCATTGG  R：TCCCTCCTGTTGCATCCATT |
| Rat-SMAD1 | F：TGGGCTGCTCTCCAATGTTA  R：GTGGTAGTTGCAGTTCCGAC |
| Rat-SMAD5 | F：CCCTGCCAATAACAAGAGCC  R：AGCCATGGTGAAAGTTGCAG |
| Rat-SMAD8 | F：CAGCATCTTTGTCCAGAGCC  R：AAAGCTCATCCGAATCGTGC |
| Rat GAPDH | F: AAGATGGTGAAGGTCGGTGT |
|  | R: TGACTGTGCCGTTGAACTTG |
| Rat U6 | F: TGCTTCGGCAGCACATATAC |
|  | R: AGGGGCCATGCTAATCTTCT |
| Rat BMPR2 | F: CTTTGCCCTCCTGCTTCTTG |
|  | R: TCGACTCTCACCAATCCCAA |
| Rat SMAD4 | F: GATCTATGCCCGTCTGTGGA |
|  | R: TGGGTTCACACAGACGCTAT |
| Rat ID3 | F: TCCGGAACTTGTGATCTCCA |
|  | R: CTGTGGCAAGATCGAAGCTC |
| **SiRNAs sequences:** | |
| si-circGSAP #1 | UGCCUCUUAUGAAGACUAUTT |
| si-circGSAP #2 | UUAUGAAGACUAUUAUGUGTT |
| si-circGSAP #3 | AUGAAGACUAUUAUGUGGCTT |
| si-BMPR2 #1 | GCCGAACUAAUUCCAAUAATT |
| si-BMPR2 #2 | GUCCACCUCAUUCAUUUAATT |
| si-BMPR2 #3 | GGGACAUAAAUCUUGUAAATT |
| **PCR primers for plasmids construction** | |
| circGSAP | F: CGGAATTCTAATACTTTCAGACTATTATGTGGCTGTTTACT |
|  | R: CGGGATCCAGTTGTTCTTACTTCATAAGAGGCAATGCAATG |
| **Mimics and inhibitor sequences** | |
| miR-27a-3p mimics | Sense (5' to 3') UUCACAGUGGCUAAGUUCCGC |
|  | Antisense (5' to 3') GGAACUUAGCCACUGUGAAUU |
| miR-27a-3p inhibitor | GCGGAACUUAGCCACUGUGAA |
| **Primers for dual luciferase activity reporter system** | |
| circGSAP-WT | F: GGGCCCGGGTCCCTGGTATTGGATTGTTG |
|  | R: GAATTCGGCAGGCAGAGACATTCTCAG |
| circGSAP-MUT | F: TAGCCCCCCCGAAGATGGCTGCGTTGCACTG |
|  | R: CGCAGCCATCTTCGGGGGGGCTAAGCAAGTGTTCTGCAGAAG |
| BMPR2- WT | F: GGGCCCTCCTGGATGGCAGCAGTATA |
|  | R: GAATTCGCGCGACTAAACAAAAGTGC |
| BMPR2-MUT | F: AACTGCCCCCCCCATGTTTTCAAGCCTATGGAGTGAAATTA |
|  | R: TGAAAACATGGGGGGGACAGTTCATTCCTATATCTTTAGACACC |
| **Sequences for probe** | |
| circGSAP | 5’Cy3-CACATAATAGTTTCATAAGAGGCA- 3’Cy3 |
| miR-27a-3p | 5'FAM-GCGGAACTTAGCCACTGTGAA |
