# Supplementary figures and images for: CircGSAP alleviates pulmonary microvascular endothelial cells dysfunction in pulmonary hypertension via regulating miR-27a-3p/BMPR2 axis
Source: Respir Res. 2022 Nov 19;23:322. doi: 10.1186/s12931-022-02248-7 (PMC9675109; doi:10.1186/s12931-022-02248-7)

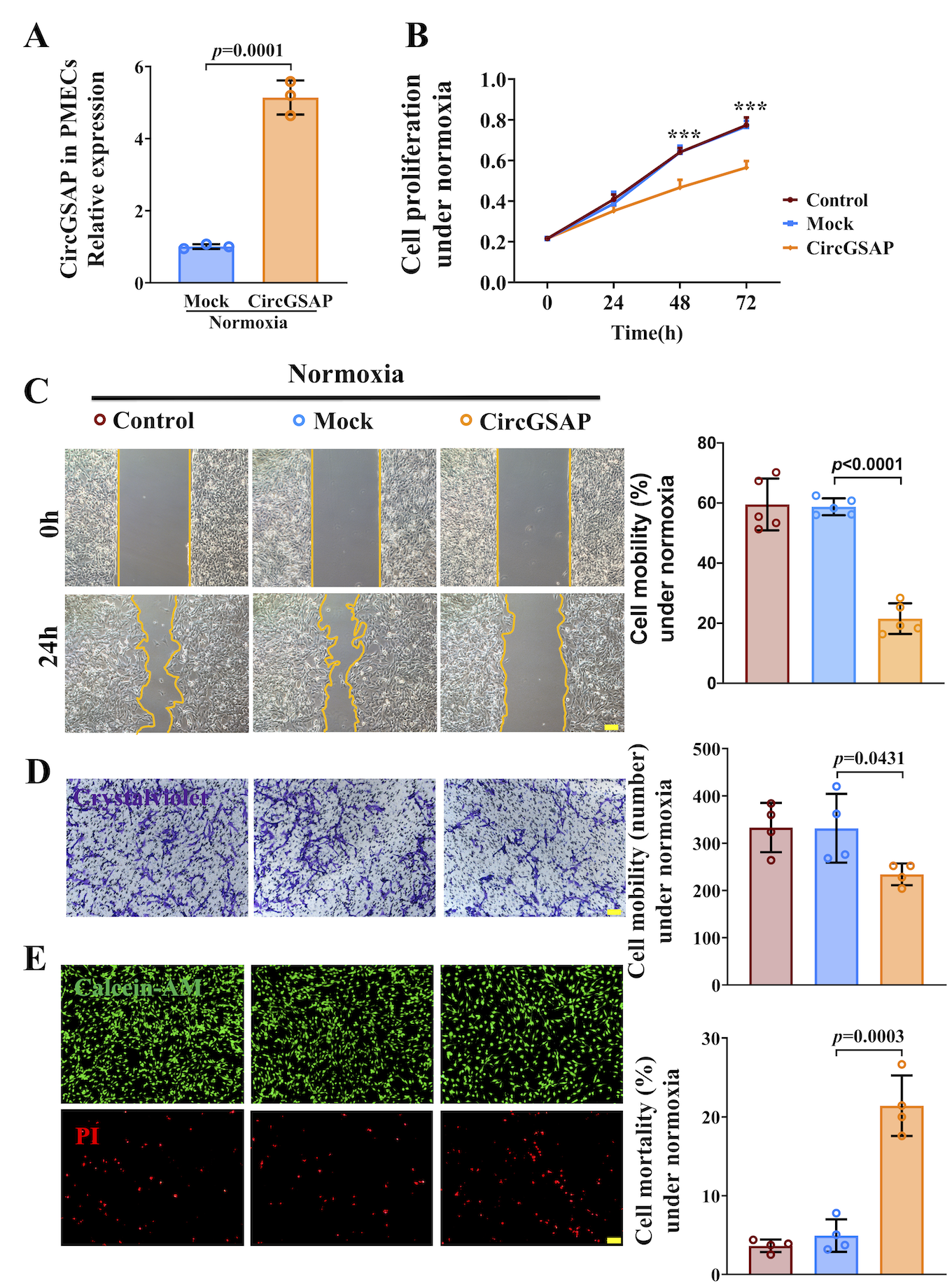

Supplement: Supplementary file 2 — Additional file 2: Fig. S1. Effects of circGSAP on the proliferation, migration and mortality of PMECs under normoxia. A Expression levels of circGSAP in PMECs treated with circGSAP under normoxia (n = 3). B-D Cell proliferation analysis, wound healing analysis and cell migration analysis of PMECs overexpressing circGSAP under normoxia (n = 5 or 4). E Cell mortality analysis of PMECs overexpressing circGSAP under normoxia (n = 4). All data are presented as the mean ± SEM. *p < 0.05; Scale bar 100 μm. **p < 0.01; ***p < 0.001. [file 12931_2022_2248_MOESM2_ESM.tiff]

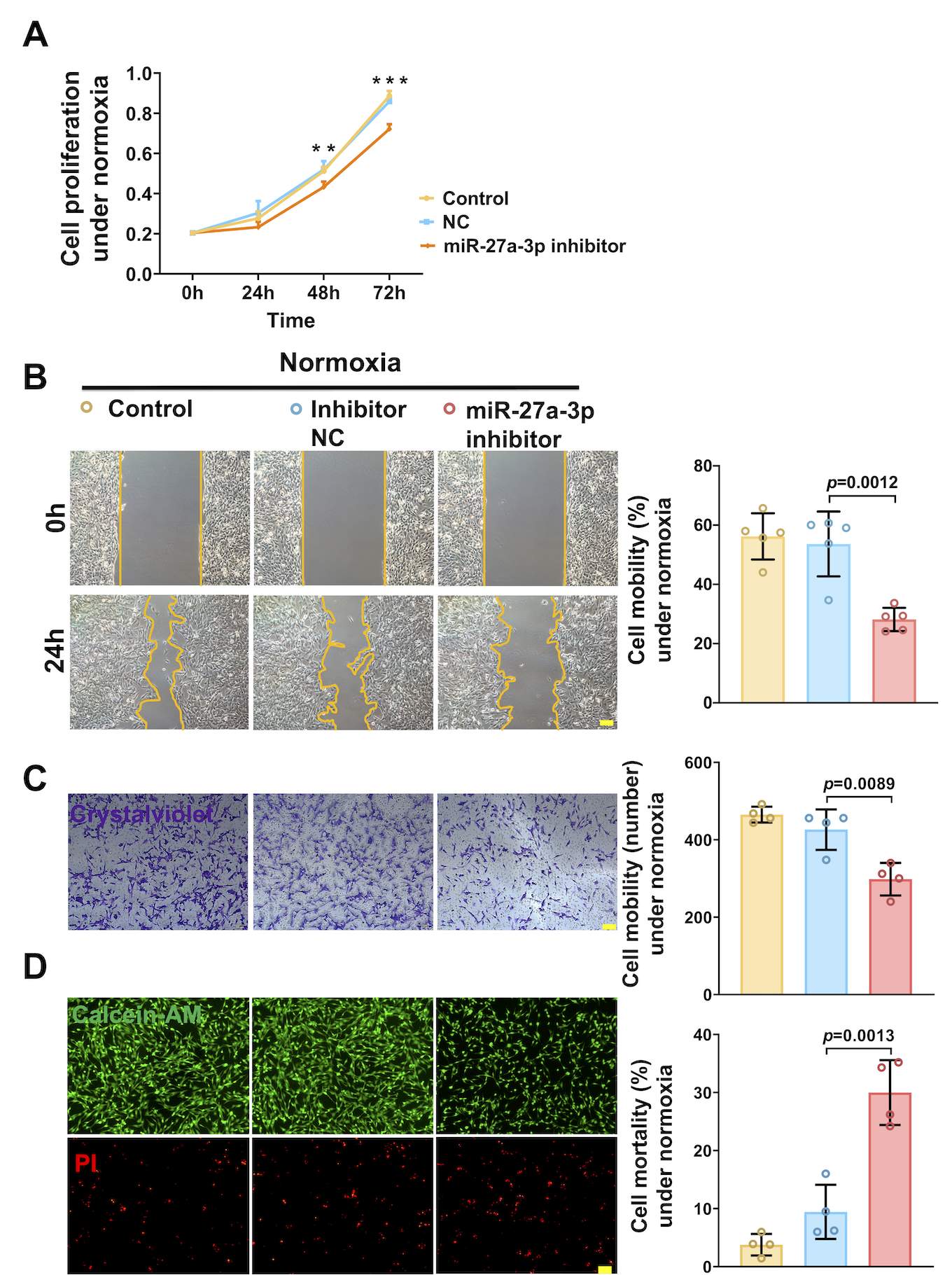

Supplement: Supplementary file 3 — Additional file 3: Fig. S2. Effects of miR-27a-3p on the proliferation, migration and mortality of PMECs under normoxia. A-D Cell proliferation analysis, wound healing analysis, cell migration analysis and cell mortality analysis of PMECs with miR-27a-3p inhibitor under normoxia (n = 5 or 4). All data are presented as the mean ± SEM. *p < 0.05; **p < 0.01; ***p < 0.001. [file 12931_2022_2248_MOESM3_ESM.tiff]
